# Two Buffalo Newspapers

**Published:** 1902-08

**Authors:** 


### Two Buffalo Newspapers.

THE Marian Murphy case which since the last issue of the JOURNAL has occupied much space in the newspapers and been the cause of many odd examples of what has received the appropriate title of "newspaper medicine," has been productive of much discussion regarding the various phases of criminology. The arrest of Charlie We, a chinese laundryman, on the charge of having murdered the child has been a mere incident in an otherwise notable case. Aside from the purely criminal phases, which to those who have given study to such matters is apparently one of those rarely discovered results of perversion, it has shown that Buffalo has a newspaper or two quite above the ordinary and which exhibited an unusual if not remarkable attitude in relation to the ethics of journalism.

Ordinarily, in such a case a mystery, with the outraged body of a child lying on a morgue slab and every newspaper hungry for news, with an eager public hanging on every issue, newspapers have much temptation to exceed the bounds of decency and present every detail to their readers. In this case *The Express* as soon as the child's body was found retained a physician to make an examination of the body and hand in a written report, which was done. The report was remarkably complete considering the difficulties to be overcome, and would have made rare reading for that portion of the public which revels in the horrors of such an uncanny crime. Instead of printing the details which it had in its possession *The Express* held it as inside information and only made use of it to further the investigations of its reporters. It should be mentioned that *The News* also had the information and it too refrained from making public use of it. Here was a rare opportunity which was decently passed by.

These newspapers exhibited uncommon self-denial in the course pursued, for they could have given to their readers all the information produced by the official post-mortem examination twenty-four hours in advance of that operation, yet they refrained from doing so for decency's sake and in a spirit of cleanliness, too often lacking in the daily newspapers at the present time. Mr. Matthews and Mr. Butler are deserving of thanks for their attitude in this matter. It is a revelation, and quite a healthy one.

